# Childhood Asthma and Environmental Exposures at Swimming Pools: State of the Science and Research Recommendations

**DOI:** 10.1289/ehp.11513

**Published:** 2008-09-30

**Authors:** Clifford P. Weisel, Susan D. Richardson, Benoit Nemery, Gabriella Aggazzotti, Eugenio Baraldi, Ernest R. Blatchley, Benjamin C. Blount, Kai-Håkon Carlsen, Peyton A. Eggleston, Fritz H. Frimmel, Michael Goodman, Gilbert Gordon, Sergey A. Grinshpun, Dirk Heederik, Manolis Kogevinas, Judy S. LaKind, Mark J. Nieuwenhuijsen, Fontaine C. Piper, Syed A. Sattar

**Affiliations:** 1 Environmental and Occupational Health Sciences Institute, Robert Wood Johnson Medical School/University of Medicine and Dentistry of New Jersey, Piscataway, New Jersey, USA;; 2 U.S. Environmental Protection Agency, National Exposure Research Laboratory, Athens, Georgia, USA;; 3 Catholic University of Leuven, Research Unit of Lung Toxicology, Laboratory of Pneumology, Leuven, Belgium;; 4 University of Modena and Reggio Emilia, Dipartimento di Scienze di Sanità Pubblica, Modena, Italy;; 5 Department of Pediatrics, Unit of Allergy and Respiratory Medicine, University of Padova, Padova, Italy;; 6 School of Civil Engineering, Purdue University, West Lafayette, Indiana, USA;; 7 National Center for Environmental Health, Centers for Disease Control and Prevention, Atlanta, Georgia, USA;; 8 Faculty of Medicine, University of Oslo, Norwegian School of Sport Sciences, Oslo, Norway;; 9 Johns Hopkins University (retired), Baltimore, Maryland, USA;; 10 Engler-Bunte-Institute, Department of Water Chemistry, University of Karlsruhe, Karlsruhe, Germany;; 11 Department of Epidemiology, Rollins School of Public Health, Emory University, Georgia, USA;; 12 Department of Chemistry and Biochemistry, Miami University, Oxford, Ohio, USA;; 13 Department of Environmental Health, University of Cincinnati, Cincinnati, Ohio, USA;; 14 Division of Environmental and Occupational Health, The Netherlands Institute for Risk Assessment Sciences, University of Utrecht, Utrecht, The Netherlands;; 15 Centre for Research in Environmental Epidemiology, Barcelona, Spain;; 16 Municipal Institute of Medical Research (IMIM-Hospital del Mar), Barcelona, Spain;; 17 CIBER Epidemiologia y Salud Pública, Spain;; 18 Medical School, University of Crete, Heraklion, Greece;; 19 LaKind Associates, LLC, Catonsville Maryland, USA;; 20 Environmental Epidemiology, Parc de Recerca Biomèdica de Barcelona, Barcelona, Spain;; 21 Truman State University (retired), Education Committee National Swimming Pool Foundation, Bushkill, Pennsylvania, USA;; 22 Centre for Research on Environmental Microbiology, Faculty of Medicine, University of Ottawa, Ottawa, Ontario, Canada

**Keywords:** aerosols, biologics, childhood asthma, DBPs, disinfection by-products, epidemiology, study design, swimming pools

## Abstract

**Objectives:**

Recent studies have explored the potential for swimming pool disinfection by-products (DBPs), which are respiratory irritants, to cause asthma in young children. Here we describe the state of the science on methods for understanding children’s exposure to DBPs and biologics at swimming pools and associations with new-onset childhood asthma and recommend a research agenda to improve our understanding of this issue.

**Data sources:**

A workshop was held in Leuven, Belgium, 21–23 August 2007, to evaluate the literature and to develop a research agenda to better understand children’s exposures in the swimming pool environment and their potential associations with new-onset asthma. Participants, including clinicians, epidemiologists, exposure scientists, pool operations experts, and chemists, reviewed the literature, prepared background summaries, and held extensive discussions on the relevant published studies, knowledge of asthma characterization and exposures at swimming pools, and epidemiologic study designs.

**Synthesis:**

Childhood swimming and new-onset childhood asthma have clear implications for public health. If attendance at indoor pools increases risk of childhood asthma, then concerns are warranted and action is necessary. If there is no such relationship, these concerns could unnecessarily deter children from indoor swimming and/or compromise water disinfection.

**Conclusions:**

Current evidence of an association between childhood swimming and new-onset asthma is suggestive but not conclusive. Important data gaps need to be filled, particularly in exposure assessment and characterization of asthma in the very young. Participants recommended that additional evaluations using a multidisciplinary approach are needed to determine whether a clear association exists.

This review derives from a workshop held in Leuven, Belgium, in August 2007 to develop a research agenda that would lead to a better understanding of children’s exposure to disinfection by-products and biologics in the swimming pool environment and whether such exposures are associated with asthma. Other health end points are of interest to those investigating swimming pool exposures but were outside the scope of the workshop.

Disinfection of swimming pools is essential to prevent outbreaks of infectious illnesses from recreational waters (Centers for Disease Control and Prevention 2007). However, traditional chemical disinfection processes result in the formation of disinfection by-products (DBPs) (Aggazzotti and Predieri 1986; Aggazzotti et al. 1998; Beech et al. 1980; Chu and Nieuwenhuijsen 2002; Fantuzzi et al. 2001; Glauner et al. 2005; Judd and Jeffrey 1995; Kim et al. 2002; Li and Blatchley 2007; Weil et al. 1980; Weisel and Shepard 1994; Zwiener et al. 2007). The specific types and levels of DBPs formed depend on numerous factors, including the type and amount of disinfectant used, characteristics of the swimming pool and pool water, and swimmer hygiene (Zwiener et al. 2007).

Swimming had been recommended as a sport for children with childhood asthma because there is experimental and observational evidence from short-term studies that swimming is less asthmagenic than other types of vigorous exercise and that asthmatics may tolerate swimming better than other types of physical activity. They may do so because of the horizontal position of the body during swimming, which alters the breathing pathway compared with other forms of exercise, or the high humidity present in indoor pools (Bar-Yishay et al. 1982; Bundgaard et al. 1982; Fitch and Morton 1971; Inbar et al. 1980; Matsumoto et al. 1999; Reggiani et al. 1988). However, increased ocular and respiratory symptoms and other adverse health end points have been reported in swimmers and attributed to exposure to disinfectants or their by-products. Adult airway hyperresponsiveness based on markers of inflammation has been reported to be more prevalent in elite swimmers than in controls or other athletes (Belda et al. 2008; Carlsen et al. 1989; Helenius et al. 1998, 2002), but not in adolescent elite swimmers (Pedersen et al. 2008). For lifeguards at swimming pools, an exposure–response relationship has been identified between trichloramine, measured as total chloramines, and irritant eye, nasal, and throat symptoms, although not chronic respiratory symptoms or bronchial hyperresponsiveness (Massin et al. 1998). Adolescent competitive swimmers reported more lower and upper respiratory symptoms and eye irritation than a group of control athletes (indoor soccer players), and the number of symptoms reported by the swimmers was related to their exposure to chloramines (Levesque et al. 2006). However, the amount of training of the swimmers exceeded that of the control group, and other end points such as wheezing during training and lifetime asthma were comparable for the two groups.

Markers of oxidative stimuli in the blood and symptoms of irritation have been also associated with higher exposure to chlorinated irritants in male swimmers 15–22 years of age (Varraso et al. 2002). The prevalence of hay fever for adults increased for those who had spent more time at swimming pools when they were children, based on a recall questionnaire (Kohlhammer et al. 2006). However, no increase in asthma prevalence or atopic dermatitis was found, although the authors indicated that the number of asthmatics may have been too small to detect an association. Outbreaks of ocular and respiratory illnesses having symptoms consistent with exposure to chloramine were reported for individuals who swam at hotel indoor pools, but the study design could not differentiate effects related to chemical agents from biological agents (Bowen et al. 2007). High chloramine levels in hotel pools were associated with more eye irritation and skin rashes (Kaydos-Daniels et al. 2008). In a case report, high air levels of chloramine have been proposed as causing occupational asthma in three pool workers (Thickett et al. 2002). Swimming pool workers in the Netherlands had more general respiratory symptoms than did the general Dutch population, and the frequency of upper respiratory symptoms, but not lifetime or physician-diagnosed asthma, was related to exposure to chloramines at swimming pools (Jacobs et al. 2007). Analyses of the Norwegian Mother and Child Cohort Study suggested that early baby swimming may be related to wheeze in toddlers, particularly for children with parental history of atopy, but the authors indicated that further investigation is warranted (Nystad et al. 2008). Kohlhammer and Heinrich (2007) reviewed published data on the relationship between chlorination byproducts in pool environments and effects on allergy and respiratory health and concluded that, for children, a trend of more frequent attendance at chlorinated pools could be an important factor in increasing frequencies of allergic disease and asthma.

Most studies of swimming pools and asthma have examined the association among adults or children of school age. However, several recent publications have explored whether exposure to respiratory irritants is associated with asthma in children who visit the indoor swimming pool environment when they are very young (Bernard et al. 2003, 2006, 2007; Carbonnelle et al. 2008; Carraro et al. 2006; Lagerkvist et al. 2004; Nemery et al. 2002; Nickmilder and Bernard 2007). Several studies have addressed pulmonary epithelium permeability based on serum biomarker levels, predictors of doctor-diagnosed asthma, elevated breath nitric oxide levels, and total and aeroallergen-specific serum immunoglobulin E (IgE) or wheezing in young children and their association with time spent attending indoor swimming pools as either schoolchildren or infants. These studies have yielded varying results (Bernard et al. 2003, 2006, 2007; Nickmilder and Bernard 2007). Swimming pool attendance in the first year of life was not associated with higher rates of atopic disease in a cohort in Germany (Schoefer et al. 2008).

Disinfection of swimming pools is most commonly achieved with chlorine. The types of chlorine generally used are sodium hypochlorite (liquid bleach), calcium hypochlorite, or chlorine gas (Ford 2007). For outdoor swimming pools, stabilized chlorine products are typically used (Ford 2007). Free chlorine (largely in the forms of hypochlorous acid and hypochlorite ion) can react with precursor materials to form DBPs. Swimming pool water contains natural organic matter precursors not only from the tap water itself but also from bathers, including constituents of sweat and urine, skin particles, hair, microorganisms, cosmetics, and other personal care products. Swimming pool DBPs may include inorganic chloramines, organic chloramines, haloacetonitriles, and other organic compounds, some of which are volatile and known respiratory irritants (Li and Blatchley 2007). Swimmers would be exposed to these DBPs, as well as the pool chemicals used as disinfectants that are irritants. There is evidence that irritant chemicals may contribute to the incidence of asthma in children and adults (McConnell et al. 2002; Medina-Ramón et al. 2005; Rumchev et al. 2004; Sherriff et al. 2005; Zock et al. 2007).

These studies have brought to the fore important questions regarding respiratory health and swimming. Several important data gaps need to be filled in order to make a definitive determination as to whether new-onset asthma in children is caused by DBPs in swimming pool air—and if so, by what mechanism. The overarching questions addressed here are whether swimming or being at indoor pools, particularly by very young children, increases the risk of new childhood asthma, and if so, what factors in the environment of the pool may be responsible.

How should studies be designed to answer these questions? The multifactorial nature of asthma etiology and the difficulties in its diagnosis, together with our fragmentary understanding of the indoor pool environment, pool maintenance, and swimmer hygiene, make these questions truly challenging to address. We focus here on data needs and best study practices for four key areas necessary for fully understanding the relationship between the pool environment and childhood asthma: *a*) distinguishing between children with asthma and children without asthma, especially young children; *b*) exposure tools for assessing factors in the pool environment potentially associated with asthma; *c*) epidemiologic research issues; and *d*) pool operation and maintenance issues affecting DBP formation. The following sections provide an overview of these four subjects together with a series of key research recommendations.

## Issues

### Correctly diagnosing asthma

Correctly diagnosing asthma is a critical but difficult aspect of investigations of associations between causation of asthma in children and exposures at indoor swimming pools. The current medical consensus definition of asthma is a chronic inflammatory disorder of the airways with an associated increase in airway hyperresponsiveness that leads to recurrent episodes of wheezing, breathlessness, chest tightness, and coughing, particularly at night or in the early morning that is often reversible either spontaneously or with treatment (Global Initiative for Asthma 2006). Asthma is a heterogeneous and multifactorial syndrome, and the clinical expression is complex. Epidemiologic studies of the association between environmental exposures, including those at indoor swimming pools, and asthma have often relied on asking if the subject has ever been diagnosed with asthma by a physician (i.e., doctor-diagnosed asthma) or symptoms reported on questionnaires to classify subjects as asthma cases or controls. The use of doctor-diagnosed asthma to assign subjects can lead to misclassifications when an individual misunderstands the physician because of a language barrier or when a physician wants to avoid labeling a patient as asthmatic because of cultural prejudice (Poureslami et al. 2007). Epidemiologic studies have different approaches and goals in determining whether a child is considered asthmatic, compared with a clinical diagnosis. The goal of clinicians is to advise and provide appropriate treatment to individual patients to forestall the onset of, cure, or manage the disease independent of the “official” diagnosis. During clinical treatment, the child is observed over time to determine response to treatment, and a variety of tests are used to improve the accuracy of the diagnosis. Epidemiologic studies for clinical and population-based research are obligated to define the disease in a rigorous, reproducible way to differentiate individuals with asthma from those without asthma, usually without the luxury of repeated observations.

There is no diagnostic test for asthma. For clinicians, a diagnosis should be based on a history of recurrent episodes of coughing or wheezing, especially when these episodes are related to typical precipitants [e.g., acute viral respiratory infections (colds), exercise, cold air] and are relieved by beta-adrenergic agents. Most clinicians would include a response to an appropriate short-acting reliever medication (albuterol by inhalation) or controller medication (inhaled corticosteroids or a leukotriene modifier) as support for the diagnosis. The presence of bronchial hyperresponsiveness by methacholine bronchial provocation or by exercise test also supports the diagnosis of asthma (Carlsen et al. 1998). A recent workgroup recommended that respiratory symptoms should not be accepted as definitive in determining whether elite (Olympic) athletes, including swimmers, have asthma, and the definition varies across countries (Fitch et al. 2008).

Epidemiologic studies of childhood swimming and new-onset asthma in children require an accurate and consistent asthma diagnosis in young children; however, diagnosing asthma in children < 3 years of age is especially difficult. Part of the difficulty is that wheezing respiratory illnesses occur very commonly in infants, and infants’ response to medication can be difficult to assess (Martinez et al. 1995). Thus, wheezing in infants, even recurrent wheezing, is not synonymous with asthma. Furthermore, although classification of wheezy infants and young children into categories of transient wheezing and persistent wheeze may be useful in the description of clinical phenotypes, they may lead to misclassification in an epidemiologic study, particularly for a retrospective assessment made later in childhood when the development of wheeze is known.

In epidemiologic studies, an assignment of “asthmatic” must be based on data that are consistently gathered, reproducible from study to study, and as quantitative and objective as possible. Most epidemiologic studies have used questions (sometimes modified) from the International Study of Asthma and Allergy in Children (ISAAC) Steering Committee (1998). The ISAAC questionnaire provides a valid tool for international comparisons of asthma symptoms in children. There is no well-validated questionnaire applicable to infants, but a validated survey for children 5–12 years of age, the Child Health Survey for Asthma, has been adapted (Asmussen et al. 1999). Questionnaires are prone to subjective evaluation and understanding by the parents. An alternate approach for assignment of categories is to use “doctor-diagnosed asthma,” although, as discussed above, the clinical definition may not provide the most consistent demarcation, particularly for the very young.

Epidemiologic studies also base assignments of asthma on pulmonary function test confirmation of obstruction. In most epidemiologic studies involving children, pulmonary function tests are limited to children ≥ 5 years of age. Thus, although pulmonary function tests are the most important confirmatory test for asthma and should be considered in all ages, it is a challenge to obtain reliable data from infants and young children (Nystad et al. 2002). However, where possible, tests such as forced or impulse oscillometry, passive respiratory mechanics, or tidal breathing flow volume loops can be useful in defining asthma.

Measures of bronchial hyperresponsiveness such as methacholine challenge, histamine challenge, exercise challenge, and mannitol inhalation have also been reported in some studies with older children (Tantisira et al. 2006; Xuan et al. 2002). Although these are objective measures for characterizing asthma, they have limited utility for diagnosing asthma, because bronchial responsiveness to methacholine is a sensitive measure of asthma but is not very specific, whereas exercise-induced bronchoconstriction is a very specific measure but has low sensitivity (Carlsen et al. 1998; Godfrey et al. 1991).

Skin prick tests or measurements of specific IgEs in serum and total eosinophils have been associated with asthma and are potentially objective tests that have been frequently included in epidemiologic studies in children (Guilbert et al. 2005). Exhaled nitric oxide (eNO) has been suggested in children > 6 years of age, but its value has not been confirmed as a diagnostic test for that age group (American Thoracic Society and the European Respiratory Society 2005). Recently, biomarkers in exhaled breath condensate (EBC) have been proposed as a tool for investigating the pathobiology of different respiratory diseases. The technique is safe and easy to perform even for children as young as 4 years of age, but its relationship to asthma, pre-asthma conditions, or early-stage changes that may or may not lead to asthma has not been validated (Horvath et al. 2005).

Information on symptoms, obtained from responses to standardized questionnaires, is considered essential to the definition of asthma (Table 1). Lung function tests are a highly desirable measure but may not be feasible in large epidemiologic studies. Among other tests that are important, those defining the atopic status of the asthmatic cases are accepted as providing the most important supporting evidence.

### Improved tools for characterizing the indoor pool environment and human exposure

#### Characterizing the indoor pool environment

It is essential to comprehensively characterize all chemicals that are potential respiratory irritants and sensitizers present in the pool air at toxicologically relevant concentrations, as opposed to merely quantifying preselected target chemicals. Exposure to putative agents needs to be determined accurately and precisely to avoid exposure misclassification leading to attenuation in risk estimates and/or loss of power. Fundamental chemistry studies on DBP formation from chlorine/organic nitrogen chemistry and alternative, nonchlorine pool treatment chemicals are needed. Basic information on aerosols and aspirated water, including distance transported, particle size distribution, and the chemical and biological composition, is currently not available and is an important aspect of the indoor pool environment used to determine exposures.

Epidemiologic studies have both suggested and questioned the proposal that DBPs, particularly trichloramine, may be at sufficiently high air concentrations within the indoor swimming pool environment to be a respiratory irritant that can induce asthma in the developing lung (Bernard 2007; Bernard et al. 2003, 2007; Nickmilder and Bernard 2007; Nieuwenhuijsen 2007). However, trichloramine has not been sufficiently studied within the pool environment to know its potential concentration range; therefore, exposure to trichloramine has not been adequately characterized to clearly demonstrate an association with asthma. Further, the most common technique used to measure trichloramine, which involves the reduction of chloramines to chlorides and analysis by ion chromatography (Héry et al. 1995), is not specific for that compound but responds to inorganic chloramines (e.g., monochloramine and dichloramine) and some organic chloramines (Li and Blatchley 2007).

In addition to chloramines, other chemical and/or biological agents present in the indoor pool environment could potentially affect the respiratory system and may contribute to an association between adverse health outcomes and time spent at indoor pools. These other agents should be identified and quantified, where possible, and investigated for allergenicity and irritant properties. For example, new research has identified other volatile DBPs, including dichloroacetonitrile and dichloromethylamine (Li and Blatchley 2007). Dichlorooxide (Cl_2_O), which is present in liquid bleach and contributes to the “chlorine” odor, warrants further investigation. Dichlorooxide can be measured with high-resolution ultraviolet spectroscopy (Cady et al. 1957), but no onsite method for indoor pool environments is available.

Table 2 lists DBPs, chemical agents, and biologics of potential interest in the pool environment that can result in irritant exposures, along with methods that can be used to measure them in water and air. Factors that can affect the levels of DBPs present in indoor pool environments and that optimally should be determined as part of any pool/DBP investigation to build exposure models (Jacobs et al. 2007) include type of disinfectant, water temperature and pH, number of swimmers, hygiene practices, the type of pool and height of the building housing it, water replacement, and the ventilation rate. Although established methods for collection and analysis exist for a number of the DBPs, improved analytical techniques are needed to establish the concentration of individual DBPs that can reach the airways, either via inhalation of gaseous agents or aerosols or via aspiration of pool water, and to understand the mechanisms and kinetics of the reactions that are responsible for their formation. Research-grade methods are important for each chemical species; however, there is also a need for simple, fast, onsite, specific, and inexpensive methods to characterize exposure for use in large epidemiologic studies and by pool operators to optimize pool conditions. Simple poolside measurement techniques are likely to respond to a broad spectrum of different chemical or biological agents and should be validated under various conditions using research-grade methods.

The possibility that biological agents may also contribute to asthma needs to be considered. Although pool water is disinfected, the warm temperatures, high humidity, and biological material regularly shed by swimmers can promote microbial contamination and growth, particularly as biofilms (Goeres et al. 2004). Swimmers’ exposures to biologics and nonvolatile species may occur from direct inhalation of aerosols or by aspiration of pool water. Rapid and accurate measurement of biologics in the pool environment can be difficult because large sample volumes (hundreds to thousands of liters) are required to determine the types and concentrations of airborne organisms and/or their components, such as endotoxins. Currently, no appropriate poolside methods are available for routine use. Potential biological targets of interest (e.g., mycobacteria and legionellae) also require special methods for sample collection and processing. Viruses, which are potential primary or co-factors for asthma in swimmers (Minor et al. 1976; Rawlinson et al. 2003), present an even greater challenge because the numbers of infectious viruses in both air and water samples are often much lower than for bacteria, thus requiring even larger sample volumes. Molecular methods, such as those based on polymerase chain reaction (PCR), can considerably accelerate the turnaround of results but cannot distinguish between live and dead microbes, severely limiting the usefulness of these methods.

Finally, associations between asthma (initiation and/or exacerbation) and use of indoor pools by children may be related to the combined influence of chemical irritants and infectious agents (Sattar et al. 2007). Indoor swimming pools constitute a unique setting with simultaneous exposure to a variety of chemicals and microbes in water and air that need to be measured with accuracy and precision to avoid exposure misclassification.

#### Characterizing human exposure

Exposure assessments in epidemiologic studies of indoor pools are often limited by the available resources, time, and methods (Nieuwenhuijsen 2003). To date, only fairly simple exposure indices have been used, including the type of swimming pool or treatment, swimming yes/no, and estimates of cumulative duration of swimming (Bernard 2007; Bernard et al. 2003, 2007; Nickmilder and Bernard 2007). More informative and validated exposure indices are needed to better evaluate potential relationships between a specific agent and the development of asthma.

Aquatic swim programs for babies and toddlers have become popular over the last few decades, and swimming has become mandatory in many school curricula. This increased use of indoor pools by the very young has increased their exposure to potential irritants within the indoor swimming pool environment. However, frequency and duration of visits and activities at indoor pools across different age groups have not been well characterized and are expected to vary across countries and cultures and within different regions of individual countries. Proper characterization of inhalation exposure requires the determination of concentrations of suspect chemicals and/or biologics in the breathing zone air, the frequency/extent of exposure, and the breathing rate of toddlers and young children in indoor pools. Assessment of activity levels is needed to evaluate the breathing rate and dose delivered to the lungs. These data are usually obtained through the use of questionnaires, which can be administered retrospectively or prospectively. The latter generally provides better exposure data, because it relies less on recall. Appendix 1 lists essential questions to be included in exposure/epidemiologic studies.

### Epidemiologic research issues

Past studies on childhood asthma and indoor swimming pools have generally relied on crude exposure indices without the use of the actual levels of putative agents, and often without adequate consideration of extraneous factors. The studies have generally been cross-sectional and have not entirely addressed the issue of reverse causality. Although research to resolve whether exposure to agents in swimming pool environments leads to the development of asthma likely requires long-term studies, insight into such an association may be gained by studying potential underlying mechanisms such as inflammation, oxidative stress, and increased lung permeability in short-term studies if likely causal agents can be identified. It is unlikely that a single study can provide the definitive answer, so several studies addressing different research questions, including several disciplines, need to be conducted together to provide the coherent evidence needed.

The specific research question will dictate the choice of study design and methodologic considerations for study implementation. For example, if the goal of a study is to provide evidence on whether or not chlorinated swimming pool attendance causes (or contributes to causing) asthma in children, a cross-sectional design is likely to be insufficient because it cannot fully address the temporal relation between asthma onset and swimming pool exposure. In those circumstances, definitive causal hypothesis testing would require a study that uses prospective cohort design. Well-designed and carefully implemented cross-sectional studies comparing prevalence of asthma or respiratory biomarkers among children with different types of activities, swimming pools, and measured exposure levels may be sufficient for hypothesis screening. These studies should record as completely as possible the lifetime history of swimming pool attendance and time of onset of asthma symptoms. If such an association is detected, it then becomes subject to more rigorous and costly tests using a more valid (preferably prospective) study design (Rothman and Greenland 1998). However, new cross-sectional studies with retrospective exposure assessment would still have inherent problems in accurately evaluating the time sequence of events and in distinguishing between incident and prevalent cases. Several prospective long-term studies are currently in various stages of planning and implementation both in the United States and in Europe (Keil et al. 2006; National Children’s Study 2007; Yuan et al. 2002). The existing birth cohorts offer an opportunity to test the hypothesis more directly; however, the availability of relevant exposure data in those studies needs to be established.

The disadvantages of long-term prospective birth cohorts are the necessarily long time before investigators are able to answer the research questions (10–15 years) and the study costs. Therefore, long-term retrospective cohort designs could be used where some of the exposure information already has been collected. For example, the large U.K.-based Avon Longitudinal Study of Parents and Children, which has enrolled 14,000 children, would provide a good study base because information on the frequency and duration of swimming and information on asthma-related outcomes have been collected regularly during childhood and sufficient information on potential confounders is available (Golding et al. 2001). For this approach, information on specific swimming pool characteristics would need to be collected and a retrospective exposure assessment conducted.

To examine short-term changes in lung function or biomarkers that may shed light on underlying mechanisms, experimental studies doing before–after comparisons or using a crossover design would likely be useful. Crossover studies could be designed to have children swimming in chlorinated pools and nonchlorinated pools (e.g., those using silver/copper, bromine, ozone, and ultraviolet) (Bernard et al., 2003) or children attending pools without swimming, in addition to including children at nonpool locations (to address the issue of potential confounding by exercise), while obtaining information on symptomatology, lung function parameters, biomarkers of inflammation, oxidative stress, and lung permeability before, during, and after exposure/exercise. These types of experiments have been successfully conducted with human exposures to agents such as diesel exhaust (McCreanor et al. 2007).

Because asthma is a multifactorial disease, any study evaluation of the association between asthma causation and hypothesized risk factors should be designed such that extraneous factors are carefully considered. Extraneous factors evaluated in asthma studies that may act as confounders or effect modifiers should include sociodemographics (urban vs. rural, socioeconomic status, ethnicity, education), number of siblings and birth order, parental smoking, family history and atopy/asthma, genetics plus dietary factors and body mass index, asthma medication (irrelevant for new onset of asthma), exercise, and past respiratory infections. Because no list of potential confounders can be considered exhaustive, it is important to ensure that *a priori* criteria that employ causal reasoning be used in deciding whether a variable is a confounder (Armstrong and Strachan 2004; Hernan et al. 2002). In general, investigators must be consistent in how they use these confounders in analyses.

Although it may be appropriate to increase precision by reducing the number of variables in a model, in doing so it is critical to avoid *a posteriori* decisions and statistical test–driven approaches (Kleinbaum et al. 2008). Also important is the use of statistical analyses correcting for multilevel sampling (Armstrong and Strachan 2004).

### Pool operation and maintenance improvements needed to reduce DBP exposures

Several important aspects of pool operation management and preventive maintenance can affect exposure to chemicals and biologics within the pool environment. Therefore, an understanding of the pool operation and maintenance practices can be an important component of epidemiologic studies that are either retrospective or do not measure exposures to all agents across the entire study population. A minimum number and frequency of water tests are needed to establish whether a pool disinfectant is maintained at the optimal levels, based on guidelines that vary across countries [e.g., for free chlorine, ideal or recommended values include 2.0–4.0 ppm, 1–2 ppm, 0.3–0.6 ppm, and 0.6–1.2 ppm for the United States, United Kingdom, Germany, and Italy, respectively; additional values for other parameters can be found in Ford (2007) and World Health Organization (2000), and further data on training within the United States is available at the National Swimming Pool Foundation (2008) website]. To facilitate optimal pool operation, recommended minimum training for all pool operators with periodic review training should be conducted. This training should include standardized methods for measuring the chemical content of water. Training should also cover the importance of proper ventilation of indoor pools. Records from these tests would help improve the exposure assessment if they were available for epidemiology studies.

Several testing methods are available to a pool operator, including colorimetric, titrimetric, turbidimetric, and electronic methods. The most common are the *N*,*N*-diethyl-*p*-phenylenediamine (DPD) test methods. At present, information is limited on which method is the best and most practical for the general pool operator and whether newer methods currently under development would yield more reliable results.

The chemistry of both well-maintained and poorly maintained pools is poorly understood. Thus, predicting the potential exposures to agents that might be linked to asthma is difficult because of the spatial and temporal variability that might exist in the pools visited by the study population. Most of the existing knowledge base was developed from wet-chemical measurements of residual chlorine, and these data are generally interpreted under the assumption that the “combined chlorine” measurement in a swimming pool is represented by inorganic chloramines (mono-, di-, and trichloramine) (Faust and Aly 1998; Wolfe et al. 1984). Specifically, the practice of “shock chlorination” calls for addition of free chlorine at a mass concentration equal to 10 times the combined chlorine signal from a wet-chemical method. This approach is based on the assumptions that inorganic chloramines can be eliminated from water through the process of breakpoint chlorination and that the combined chlorine residual is present in the form of inorganic chloramines. In breakpoint chlorination, inorganic chloramines are completely oxidized to nitrogen, and chlorine is simultaneously reduced to chloride (Faust and Aly 1998; Wolfe et al. 1984). For example, for monochloramine,


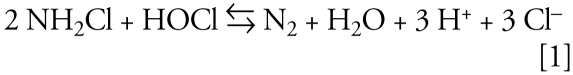


However, the “combined chlorine” in swimming pools can often be dominated by organic chloramines (Li and Blatchley 2007) and the responses of these compounds to existing shock chlorination practices are largely unknown. These generalizations apply to analyses of water samples from well-maintained pools, where the true inorganic chloramine concentration was quite low, and all quantifiable inorganic combined chlorine was present in the form of trichloramine. It is likely that the combined chlorine residuals in poorly maintained pools will be more complex. Understanding how practices affect the production of DBPs can guide the development of study questions that would improve the exposure assessment portion of epidemiologic studies.

## Conclusions and Research Recommendations

Current evidence of an association between visits to indoor pools by babies and young children and new-onset asthma is suggestive but not conclusive. Shortcomings exist in current studies in the areas of exposure assessment, the age at time of exposure, and the characterization of asthma. In particular, there is a lack of knowledge regarding the levels of DBPs, other chemicals, and biological respiratory irritants and the appropriate air collection and measurement methodologies. Pool construction and maintenance are key to minimizing exposure to irritants, but the percentage of pools maintained optimally is unknown. Independent of any association of exposures in indoor pools and asthma, procedures to optimize pool operation and maintenance should be followed and improved upon. Below we give specific study recommendations for each of the four key areas: asthma, exposure, epidemiology, and pool operation and maintenance.

### Asthma

- Use harmonized definitions of asthma across epidemiologic studies with the ISAAC questionnaire as a basis for defining asthma cases.
- Carefully evaluate new biomarkers that may prove useful in epidemiologic studies (e.g., eNO, EBC) for demonstrating potential lung epithelial damage and other relevant end points.

### Exposure

- Comprehensively identify and quantify chemicals (inorganic and organic) in the indoor pool air (especially just above the water surface) that occur at toxicologically relevant concentrations, and determine the spatial and temporal variation of key chemicals for a range of pool environments. As toxicologic information is obtained, focus exposure measurements on those chemicals that cause asthma/irritant effects at environmentally relevant concentrations.
- Determine which groups of chemicals are detected by conventional poolside methods (e.g., the DPD method) and compare these results to more sophisticated, comprehensive methods to understand which individual chemical species are included in the combined chlorine data or other measurements.
- Develop an exposure characterization for aerosols, aspirated pool water, and the gas phase for chemical and biological agents based on air concentrations and age- and culture-specific characteristics that include attendance at pools, swimming/playing activities, and breathing rates within the pool environment. Determine how pool maintenance and operation can affect the exposure, and establish which aspects of those practices should be determined in epidemiologic studies to improve the exposure estimate.
- Test already-identified and newly recognized agents detected in indoor pool environments for allergenicity and irritant properties (cellular studies, toxicologic studies) and define dose–response characteristics at relevant ambient exposure doses.

### Epidemiology

- Conduct pilot studies on questions regarding childhood/baby swimming to be used in cohort studies; gather information on temporal variability and pool-type variability to develop a uniform questionnaire; develop robust exposure indices for respiratory irritants and allergens present at indoor swimming pools; and evaluate the utility of short-term changes in biomarkers and lung function tests on the underlying mechanism of asthma causation by respiratory irritants.
- Determine whether existing prospective birth cohort studies could be used to evaluate the potential relationship between exposure to chemicals and biologics at indoor pools and the development and progression of childhood asthma.
- Replicate cross-sectional studies in different settings, including an evaluation of the time sequence of events (onset of asthma; swimming in pools), and consider recruiting children using chlorinated pools and children using pools with other types of disinfectants. Also consider studies using a before–after design or a crossover design.

### Pool operation and maintenance

- Collect data on pool disinfection and maintenance conditions by pool operators and how this alters the production of DBPs and biologics to better predict exposure for use in future epidemiologic studies and in the short term to determine how to reduce DBPs at indoor swimming pools.
- Provide better education and improved test methods for pool operators on pool chemistry to promote understanding the chemical consequences of overdosing or underdosing of pool water. This should include education and outreach materials (along with social science research) for improving personal hygiene for pool attendees (e.g., showering with soap before swimming, no urinating in pool).
- Make available a Web-based clearinghouse for people with demonstrated problems associated with municipal swimming pools.

The issue of childhood swimming and new onset of childhood asthma is complex and has clear implications for public health. If swimming at indoor pools increases the risk of childhood asthma, then concerns are warranted and action is necessary. On the other hand, if there is no relationship between childhood swimming and asthma onset, then concerns would be unwarranted and could unnecessarily deter children from indoor swimming and/or compromise water disinfection. Addressing this issue will require the close collaboration of environmental engineers, inorganic and organic chemists, exposure scientists, microbiologists, toxicologists, epidemiologists, pool maintenance experts, and physicians specializing in pediatric and adult respiratory disease.

## Figures and Tables

**Table 1:** Recommended symptoms and tests of lung function for use in epidemiologic studies on asthma in children.

Age (years)	Symptoms	Lung function	Supplemental or exploratory evaluations
All ages	Three or more episodes of bronchial obstruction (characterized by wheeze, dyspnea, physical signs of distress) Highly desirable to have medical exam confirmation	Pulmonary function tests (FEV_1_, FEF_50_) Objective evidence of obstruction Evidence of reversibility (e.g., 12% improvement of FEV_1_ after albuterol inhalation)	Extraction of medical records Evidence of atopy (skin prick tests or CAP RAST) Elevated peripheral blood eosinophils Atopic illness (eczema or food allergy)
1–3	Three or more episodes of obstruction Highly desirable to have medical exam confirmation	Tidal breathing flow:volume loops Forced impulse oscillometry Passive respiratory mechanics	Evidence of atopy
3–6	Intermittent wheezing (see all ages definition) Persistent wheezing (wheezing other than with colds)	Forced oscillometry Interrupter technique	Evidence of atopy eNO^a^ Inflammatory markers in EBCs^a^
≥ 6	Intermittent asthma Persistent asthma Doctor-diagnosed asthma	Spirometry Plethysmography Evidence of reversibility	Evidence of atopy eNO^a^ Inflammatory markers in EBC^a^ Bronchial hyperresponsiveness (methacholine, exercise, mannitol inhalation)

Abbreviations: CAP RAST (radioallergosorbent test) refers to the ImmunoCAP Specific IgE blood test (Pharmacia Diagnostics AB, Uppsala, Sweden); exam, examination; FEF_50_, forced expiratory flow at 50% of vital capacity; FEV_1_, forced expiratory volume in 1 sec.

a Currently, published data are too limited to recommend these tests as essential to defining asthma.

**Table 2:** Agents, including DBPs, in indoor pools and measurement methods for air and water and additional chemicals and biologics (and analytical methods) for which data are needed to fully understand the pool environment.

Chemical	Analytical method(s)
Primary agents	
Trichloramine (NCl_3_)	Héry et al. (1995) method (nonspecific)
Dichloramine (NHCl_2_)	DPD (nonspecific)
Monochloramine (NH_2_Cl)	MIMS
	Small portable mass spectrometer

Free available chlorine (HOCl)	DPD

Chlorine gas (Cl_2_)^a^	OSHA ID-101^b^
	NIOSH 6011^c^

Cyanogen chloride (CNCl)	GC-ECD GC/MS
Dichloromethylamine (CH_3_NCl_2_)	MIMS
Dichloroacetonitrile	

Dichlorooxide (Cl_2_O)	High-resolution UV spectroscopy
Additional agents	
Volatile organic halogen (VOX)	P&T GC-ECD or GC-MS

Bromate (BrO_3_^−^)^d^	IC conductivity
Chlorate (ClO_3_^−^)^d^	IC-MS
Chlorite (ClO_2_^−^)^d^	

Cyanuric acid^d^	GC-ECD, GC/MS
Biologics	
Bacteria (e.g., environmental mycobacteria, legionellae, pseudomonads, *Streptococcus pneumoniae*, *Haemophilus influenzae*)	PCR

Fungi (e.g., *Aspergillus niger*, *Candida* spp., *Trichophyton* spp.)	*In vitro* culture

Viruses (e.g., rhinoviruses, adenoviruses, enteroviruses)	Isolation and quantitation in cultured susceptible cells from mammalian hosts

Protozoa [free-living amoebae that *a*) can infect humans directly from water and *b*) may carry bacterial pathogens, e.g., legionellae]	*In vitro* culture

Endotoxins	*Limulus* amebocyte lysate test

Nasal mucous and nasal epithelium	Nasal swabs

Abbreviations: DPD, *N*,*N*-diethyl-*p*-phenylenediamine; ECD, electron capture detection; GC, gas chromatography; IC, ion chromatography; MIMS, membrane introduction mass spectrometry; MS, mass spectrometry; NIOSH, National Institute for Occupational Safety and Health; OSHA, Occupational Safety and Health Administration; P&T, purge and trap; UV, ultraviolet.

a Measurement techniques applicable only to air.

b OSHA (1991).

c NIOSH (1994).

d Likely to be present in water phase and not in air.

## Appendix 1

We recommend that the following essential concepts be included as part of questionnaire and data collection for epidemiologic studies to assess exposure to chemicals and biologics at indoor swimming pools:

### Information to obtain from participant

- Whether the subject is asthmatic (see Table 1 for approaches)
- Age when child first went to indoor swimming pools (whether or not for swimming)
- For different age categories (suggest 0–2, 3–5, 6–8, and = ≥8 years of age) obtain the following:
- — Number of visits per week, average duration of visits, number of visits per year, how crowded the pool was during use
- — Whether and what types of odors were noticeable (particularly “chlorine” smell)
- — What type of disinfectant was used (e.g., chlorine, ozone, ultraviolet lamp, other, unknown)
- — Type of pool used and reason for attendance, along with percentage of time for each (e.g., attend with class, competitive swimming, recreational pool, child’s vs. adult pool, private home pool, municipal pool, private club pool, other).

### Information to obtain about pool facilities (either participant or facility manager)

- Number and type of pools present (e.g., child, lap pools, adult swimming–playing, mixed use, whirlpool, Jacuzzi, water play area), additional water features (e.g., slides, fountains, rides).

### Information to obtain from pool facility

- Typical water and air temperature (for years of interest), size of room containing pool(s), depth of each pool, type of disinfectant used, frequency of disinfection (routine, shock), type/size of showering facility and if showering is mandatory before pool use (and how this is enforced), average number of swimmers in pool (per day, per year), specific times of year the pool is closed, typical free chlorine residual and combined chlorine residual for time period of interest and whether it has changed (milligrams per liter as Cl_2_), shock chlorination regime (how much chlorine is added?).
